# Authors’ Response to Peer Reviews of “Prevalence and Determinants of Academic Bullying Among Junior Doctors in Sierra Leone: Cross-Sectional Study”

**DOI:** 10.2196/75127

**Published:** 2025-05-22

**Authors:** Fatima Jalloh, Ahmed Tejan Bah, Alieu Kanu, Mohamed Jan Jalloh, Kehinde Agboola, Monalisa M J Faulkner, Foray Mohamed Foray, Onome T Abiri, Arthur Sillah, Aiah Lebbie, Mohamed B Jalloh

**Affiliations:** 1 College of Medicine and Allied Health Sciences, University of Sierra Leone, Freetown, Sierra Leone; 2 Department of Public Health, Chamberlain College of Health Professions, Chicago, IL, United States; 3 University of Sierra Leone Teaching Hospitals Complex, Freetown, Sierra Leone; 4 College of Health Sciences and Public Policy, Walden University, Minneapolis, MN, United States; 5 School of Public Health, University of Washington, Seattle, WA, United States; 6 Faculty of Health Sciences, Department of Medicine, McMaster University, 1280 Main Street West, Hamilton, ON, L8S 4L8, Canada, 1 9059622812

**Keywords:** academic bullying, junior doctors, Sierra Leone, mental health, professional development

*This is the authors’ response to peer-review reports for “Prevalence and Determinants of Academic Bullying Among Junior Doctors in Sierra Leone: Cross-Sectional Study.”*

## Round 1 Review

### Reviewer AQ [1]

#### Specific Comments

##### Major Comments

###### Introduction

*I think the Introduction in this study [* *2* *] needs to be contextualized properly. Saying that bullying in the health care profession has not been looked at is largely correct, but the authors need to strengthen their argument by properly discussing the current literature on bullying in the Sierra Leone educational establishment and the limitations of the current literature as it relates to their topic of enquiry.*

*Please read the following:*

- *Osborne A, James PB, Bangura C, Tom Williams SM, Kangbai JB, Lebbieie, A. Bullying victimization among in-school adolescents in Sierra Leone: a cross-sectional analysis of the 2017 Sierra Leone Global School-Based Health Survey. PLOS Glob Public Health. Dec 22, 2023;3(12):e0002498. [doi: 10.1371/journal.pgph.0002498] [PMID: 38134001]*
- *Report on findings from school-related gender-based violence action research in schools and communities in Sierra Leone [* *3* *].*

**Response:** We thank the reviewer for their helpful feedback and suggested references. We have revised and expanded the Introduction section with suggested references (see pages 4 and 5).

###### Methods

*I wonder why the authors decided not to recruit all junior doctors who met their inclusion criteria, given that the list of junior doctors in the University of Sierra Leone Teaching Hospitals Complex at the time of data collection can be obtained from each of the constituent teaching hospitals. I know for a fact that the population of junior doctors is not so huge (less than 500). In other words, why did the authors just recruit all 160 junior doctors? Such data can be sourced from the Sierra Leone Medical and Dental Association or from the respective teaching hospital.*

**Response:** Thank you for highlighting this important point. We recognize that the total population of junior doctors at these facilities is indeed under 500. Our original intention was to recruit all eligible junior doctors, which would have strengthened the study’s power and rendered sample size calculations less critical. However, achieving a 100% response rate proved difficult—particularly given the 3- to 6-month rotation schedules that complicate maintaining an up-to-date sampling frame. Consequently, we used a pragmatic sampling strategy, distributing the survey through the Sierra Leone Medical and Dental Association forums and across the respective hospitals for several weeks. While this approach did not capture every potential respondent, it yielded a sufficiently robust sample to draw meaningful conclusions despite the inevitable limitations of incomplete participation.

*What informed the design of the questionnaire used? Why did the authors decide not to conduct any form of validation of the questionnaire (ie, externally or internally) to ensure it is appropriate for the context in which it is used?*

**Response:** Thank you for your insightful questions regarding the questionnaire design and validation. Our questionnaire was primarily informed by prior studies from the subregion—most notably the work by Afolaranmi et al [4] in Nigeria, whose clinical training context is highly comparable to Sierra Leone. Given that many Sierra Leonean medical educators and clinical trainers received their training in Nigeria and a number of Nigerian professors practice in Sierra Leone, we found these instruments to be a suitable starting point.

To enhance contextual relevance, we conducted a pilot with 10 participants to assess clarity, applicability, and cultural appropriateness prior to rolling out the full study (see page 7). However, we acknowledge the lack of a psychometric validated tool in the manuscript’s Limitations section.

*This study was among junior doctors, but the authors mentioned registrars. A registrar is no longer a junior doctor. I may be wrong, but I strongly suggest that the authors provide a clear definition of what is the definition of junior doctor in Sierra Leone.*

**Response:** Thank you for raising this important clarification. In many settings, the term “registrar” refers to a physician who has moved beyond the intern or house officer stage and may be considered more senior. However, in the context of Sierra Leone’s postgraduate training system, registrars still fall within the broader category of early-career physicians, who have not yet obtained final specialist accreditation.

To be specific, a “junior doctor” in Sierra Leone typically includes:

- House officers/interns, who have recently graduated and are completing supervised practice
- Medical officers, who work more independently but have not pursued formal residency training
- Registrars (residents), who are enrolled in specialty training programs and have not yet become fully accredited specialists

This aligns with the general World Medical Association perspective that “junior doctors” encompass physicians in postgraduate training who have not yet achieved final specialty qualification. In Sierra Leone, this definition covers registrars, as they remain in an active training pathway and do not possess full consultant status. Hence, our study included registrars under the umbrella of “junior doctors.” We hope this clarifies why registrars were incorporated into our sample.

###### Discussion

*I beg to disagree. A sample was calculated, and a probabilistic sampling method was used in this study, which means that it gives an equal chance for everyone to be chosen. Thus, the sample used is representative of junior doctors in the University of Sierra Leone Teaching Hospitals Complex. There are two ways to explain your finding: either the sample is not representative because the sampling was not probabilistic or the whole population should have been recruited, or the finding is correct (ie, there are no gender differences).*

**Response:** Thank you for your insightful feedback. We fully acknowledge that our study was designed with a calculated sample size and a probabilistic sampling method, with the aim of ensuring a representative sample of junior doctors in the University of Sierra Leone Teaching Hospitals Complex. This design typically affords every eligible participant an equal opportunity to be selected. Thus, our finding of no statistically significant gender differences in bullying could indeed reflect a true lack of disparity within this specific population.

We appreciate your perspective and have revised the Discussion to more clearly articulate these points.

### Minor Comments

*The first two sentences of the third paragraph of the Introduction section: This has already been stated in the previous paragraph. This is just a repetition.*

**Response:** Thank you. We have revised the “Introduction” section with suggested changes (see pages 4 and 5).

## Round 2 Review

### Reviewer EN [5]

#### General Comments

*This study presents a survey of junior doctors in Sierra Leone hospitals and their experience of bullying and found high levels of bullying among the participants. Below are comments and suggestions for clarifying and strengthening the work.*

#### Specific Comments

##### Major Comments

*1. The author’s definition of bullying and whether it was provided to participants is somewhat unclear. In the abstract, bullying is described as involving repeated behaviors, which aligns with the typical definition of bullying as an ongoing or repeated action. However, in the Methods section, participants were asked to respond based on any instance of various behaviors. While a single act of intimidation, for example, constitutes inappropriate behavior that should be addressed, it may not meet the standard definition of bullying. It is essential to clarify this distinction and ensure that participants also recognized the difference so that general poor behavior is not conflated with bullying.*

**Response:** Thank you for emphasizing this point. Our study was conducted using the recognized definition of bullying as involving repeated behaviors. In our original design and implementation, we informed participants that bullying typically denotes a pattern of ongoing or repeated actions. We acknowledge, however, that some of our language in the manuscript may have led to confusion around single versus repeated incidents. We have therefore reviewed and refined our wording throughout the text—particularly in the abstract and Methods section—to ensure consistent use of the term “bullying” and to clarify that isolated, one-time acts, while concerning, may not meet the standard definition of repeated harmful behavior (see page 7).

*2. Was sampling randomly, equally, or proportionally distributed across the four sites, and were there any analyses done based on site?*

**Response:** Our sampling was designed to be random at the individual level rather than equally or proportionally allocated to each site. Because junior doctors rotate across the four sites at the University of Sierra Leone Teaching Hospitals Complex, we treated all eligible doctors as a single sampling frame. Each individual had an equal probability of selection through a computer-based random procedure, independent of their current site.

Regarding site-level analyses, we elected not to perform them because the frequent rotations diminished the value of comparing departments as distinct groups. Instead, we focused on the overall experiences of junior doctors within the hospital complex. Any subgroup analysis by site would have been confounded by the high degree of overlap in personnel across the four locations (see page 6).

*3. How was random sampling achieved?*

**Response:** Thank you for highlighting this important methodological detail. We ensured that each eligible junior doctor had an equal probability of being included by employing a computer-based random selection procedure. Specifically:

- Comprehensive sampling frame: We first compiled a roster of all junior doctors who met our eligibility criteria (aged ≥18 years and employed at the University of Sierra Leone Teaching Hospitals Complex for ≥6 months).
- Unique identifiers: Each individual in this roster was assigned a unique numeric code.
- Random number generation: We then used a random number generator to select participants based on their assigned numeric codes, thereby ensuring that every eligible junior doctor had the same chance of selection.

This approach was chosen to reduce selection bias and maintain methodological rigor, despite the logistical challenges posed by junior doctors’ frequent rotations across departments (see page 6).

*4. Please comment on the reliability and validity of the instrument used to collect data. What literature was used to inform the development of the questions? Please include this information in the manuscript.*

**Response:** Thank you for your insightful questions regarding the questionnaire design and validation. Our questionnaire was primarily informed by prior studies from the subregion—most notably the work by Afolaranmi et al [4] in Nigeria, whose clinical training context is highly comparable to Sierra Leone. Given that many Sierra Leonean medical educators and clinical trainers received their training in Nigeria and a number of Nigerian professors practice in Sierra Leone, we found these instruments to be a suitable starting point.

To enhance contextual relevance, we conducted a pilot with 10 participants to assess clarity, applicability, and cultural appropriateness prior to rolling out the full study (see page 7). However, we acknowledge the lack of a psychometric validated tool in the manuscript’s Limitations section.

*5. At the start of paragraph 3 of the Introduction, the authors refer to “other contexts”; it is unclear what contexts are being referred to in this and the preceding paragraph.*

**Response:** We thank the reviewer for their helpful feedback and suggested references. We have revised and expanded the Introduction section, including suggested references by another reviewer (see pages 4 and 5).

*6. The Introduction and Discussion would be strengthened by more specific references to literature findings. I found the text in both a little superficial.*

**Response:** We thank the reviewer for their helpful feedback. We have revised and expanded the Introduction and Discussion sections, including suggested references by another reviewer (see pages 4, 5, and 11‐15).

*7. It is unclear whether the participants were reporting behaviors they personally experienced (ie, they were bullied) against behaviors they observed (ie, others being bullied).*

**Response:** We specifically designed our questionnaire to capture bullying events that respondents personally experienced, rather than those they witnessed. The survey items regarding workplace bullying were phrased to reflect direct, firsthand encounters. Respondents who indicated experiencing bullying were then asked to describe the nature of these incidents, ensuring the data represented self-reported victimization rather than secondhand observations (see page 7).

*8. Please provide clarification as to who is a “junior doctor.” This journal has an international readership, and this term can be used differently in different countries, with “junior doctors” having different lengths of service. Please ensure this is clear within the body of the manuscript.*

**Response:** Thank you for noting this. In Sierra Leone, the term “junior doctor” encompasses three main groups:

- House officers/interns: recently graduated doctors in a period of closely supervised practice
- Medical officers: physicians who have completed internships and can work more independently but have not pursued formal residency training
- Registrars (residents): doctors actively enrolled in specialty training programs who have not yet attained full consultant (specialist) status

This aligns with the broader World Medical Association definition, which frames “junior doctors” as physicians in postgraduate training who have not yet achieved their final specialty qualifications. We have included all three categories in our study, as they each fulfill the criteria of postgraduate training without full specialist accreditation (see pages 5 and 6).

*9. The description of the multiple regression seems a little excessive given the lack of statistical significance. This could be made more concise and simply refer readers to Table 3. Similarly, the authors should be cautious not to overemphasize these findings.*

**Response:** Thank you for this valuable feedback. We appreciate the concern about potentially overstating findings that did not reach statistical significance. We believe it is important to retain the full results for completeness and transparency—even when no statistically significant associations emerge. In light of your suggestion, we will ensure that our manuscript clearly indicates the nonsignificant nature of these results and refrain from overemphasizing their importance in the Discussion.

*10. The list of references needs to be reviewed to ensure that all items have full bibliographic details.*

**Response:** Thank you for noting this. We have carefully reviewed and updated the reference list to ensure that all citations include complete bibliographic details.
